# Spatial heterogeneity determines the gastrointestinal microbiome signatures and ecological processes that govern bacterial community assembly in sheep

**DOI:** 10.1128/spectrum.01110-24

**Published:** 2024-12-23

**Authors:** Yukun Zhang, Jiangbo Cheng, Changchun Lin, Fadi Li, Xiaoxue Zhang, Chong Li, Deyin Zhang, Xiaobin Yang, Dan Xu, Yuan Zhao, Liming Zhao, Xiaolong Li, Huibin Tian, Xiuxiu Weng, Weimin Wang

**Affiliations:** 1 State Key Laboratory of Herbage Improvement and Grassland Agro-ecosystems; Key Laboratory of Grassland Livestock Industry Innovation, Ministry of Agriculture and Rural Affairs; Engineering Research Center of Grassland Industry, Ministry of Education; College of Pastoral Agriculture Science and Technology, Lanzhou University, Lanzhou, China; 2 Institute of Animal Science, Xinjiang Academy of Animal Sciences, Urumqi, China; 3 College of Animal Science and Technology, Gansu Agricultural University, Lanzhou, China; University of California, Davis, San Bernardino, California, USA

**Keywords:** sheep, gastrointestinal tract, co-occurrence networks, enterotypes, bacterial community assembly

## Abstract

Sheep are one of the globally significant livestock, providing meat, dairy products, and wool for human life, playing an indispensable role in human civilization. Despite significant advancements in microbiome research in recent years, most studies have focused solely on the rumen, lacking a comprehensive study covering the microbiome of different gastrointestinal tract (GIT) regions in sheep. In this study, we collected 338 samples from 10 different regions of the sheep GIT and systematically investigated their microbiome signatures, including community structure, enterotypes, interactions among taxa, and microbial community assembly. Our results showed that the bacterial diversity of sheep GIT exhibited a U-shaped pattern along the GIT, with the lowest diversity in the jejunum. The bacterial community composition and enterotype varied along the GIT, mainly divided into three distinct groups (four-chambered stomach, small intestine, and large intestine). The rumen had the highest total number of bacterial taxa, unique taxa, and unique functions, while the enterotypes were the same in the three regions of the large intestine. The bacterial co-occurrence networks differed greatly between different GIT regions, with more positive correlations than negative ones. Furthermore, we found that the assembly processes of bacterial communities in the four-chambered stomach and small intestine were mainly stochastic, while those in the large intestine were mainly shaped by deterministic processes, with a higher ecological niche width than other GIT regions. Our results reveal the spatial pattern of bacterial communities in the sheep GIT and the intrinsic mechanisms of bacterial community assembly, laying the foundation for microbial interventions to improve sheep productivity and sustainable farming.

**IMPORTANCE:**

Sheep's gastrointestinal tract harbors a diverse microbial community crucial for immune system balance, nutrient digestion, and overall health. We explored the microbial community composition, community types (enterotypes), bacterial interactions, and ecological processes in 10 gastrointestinal regions of 36 six-month-old Hu sheep raised under same diets and environmental conditions. Our findings revealed a unique U-shaped pattern of bacterial diversity from the rumen to the rectum, with the lowest diversity in the jejunum. The composition and enterotypes of bacterial communities varied spatially along the gastrointestinal tract, primarily categorized into three distinct groups. The rumen exhibited the highest abundance of bacterial taxa, unique taxa, and unique functions, while the enterotypes in the three regions of the large intestine were consistent. We explored the assembly processes of bacterial communities, elucidating how they find their ecological niches based on their characteristics and environmental demands. The assembly processes in the four-chambered stomach and small intestine resembled random selection, where bacterial positioning depended on luck and chance, while in the large intestine, it appeared more deterministic, with specific bacteria likely selected based on their unique skills and environmental requirements. This study enhances our understanding of microbial coexistence and interactions in complex ecosystems, with implications for improving animal productivity, disease treatment, and the development of novel microbial formulations.

## INTRODUCTION

China is a major sheep-raising country, with the highest sheep inventory, breeding stock, and meat production in the world (1). Due to the absence of religious taboos and its unique flavor, sheep meat has become one of the most popular meats in China, and its consumption is increasing (2). Hu sheep is the most popular native breed of sheep in China, known for its perennial estrus, high fecundity, good lactation performance, and strong adaptability (3). Good performance, excellent flavor, low fat content, and high feed efficiency have become the new direction of modern sheep meat production. However, the performance of Hu sheep is lower than that of other imported breeds (4, 5). In addition, global environmental issues have accelerated the change in traditional feeding methods, and sheep farming is transitioning from traditional grazing to indoor feeding (6). Due to these changes, the breeding program of Hu sheep needs to be optimized to meet the growing market demand.

In recent years, it has been discovered that the composition of the sheep gastrointestinal tract (GIT) microbiome is related to many host attributes and performance. In particular, the relationships between the GIT microbial ecosystem and sheep host immunity (7), body weight (8), and feed efficiency (9) are typical examples of the host performance-microbiome relationship. However, these studies, taking into account the characteristics of ruminant digestion and non-invasive sampling and repeatability, have only explored the relationship between microbiota and hosts in a single GIT region (especially the rumen and feces), ignoring the attention to the interpretation of the relationship between different GIT regions. The impact of different GIT regions on community structure has also been less mentioned. The wide differences in environmental heterogeneity and of intestine between different GIT regions lead to the settlement of microbial communities flowing with food in different GIT regions, playing different functions, and thus leading to different microbial-host interaction patterns in different GIT regions (10). Therefore, exploring the spatial heterogeneity of the entire GIT region is important for further improving the efficient utilization of Chinese native sheep feed and forage.

Understanding the ecological processes of GIT microbial community assembly helps to identify how the composition of GIT microbial communities responds to various changes. Deterministic and stochastic processes, based on ecological niche theory and neutral theory, are commonly used to explain microbial community assembly (11). Deterministic processes include non-biological factors (pH, temperature, etc.) and biological factors (predation, symbiosis, etc.) (12), which determine the presence and relative abundance of taxa and are related to ecological selection. Stochastic processes include unpredictable disturbances, probabilistic dispersal, and random birth-death events, which are not adaptive results determined by the environment (13). Deterministic and stochastic processes work together to regulate community assembly in ecological communities (14, 15). For example, Feng et al. studied the assembly of the entire intestinal bacteria during chicken development and demonstrated that the intestinal bacteria of chickens are mainly shaped by deterministic processes of homogeneous selection and stochastic processes of homogenizing dispersal; with the development of chickens, the deterministic effect controlling the assembly of the cecum bacteria gradually increases (16). Although a large amount of knowledge has been accumulated in bacterial community assembly research, it has been focused on ecological environment research. We know very little about the relative contributions of different ecological processes to different GIT microbiomes in sheep, which is crucial for predicting the role of microbes in regulating host-microbe ecosystem functions.

This study focuses on Hu sheep raised under the same conditions, using high-throughput 16S rRNA sequencing technology to analyze the characteristics of bacterial diversity, enterotype, and niche breadth of 10 GIT bacterial communities along the sheep GIT. At the same time, the complexity and stability of microbial ecological networks in different GIT regions were explored, and the ecological assembly processes of microbial communities in different GIT regions of sheep were analyzed. It is hypothesized that the GIT region will significantly affect the diversity, interaction, enterotype, niche breadth, and ecological assembly processes of sheep GIT microbiota. This study constructed a relatively complete biogeographical map of Hu sheep and explored for the first time the assembly mechanism of bacterial communities in all GIT regions of sheep, which helps to reveal the interaction mechanism between sheep GIT microbiota and hosts, providing an opportunity to explore microbial manipulation methods to improve sheep performance.

## MATERIALS AND METHODS

### Animals and sample collection

**Fig 1:**
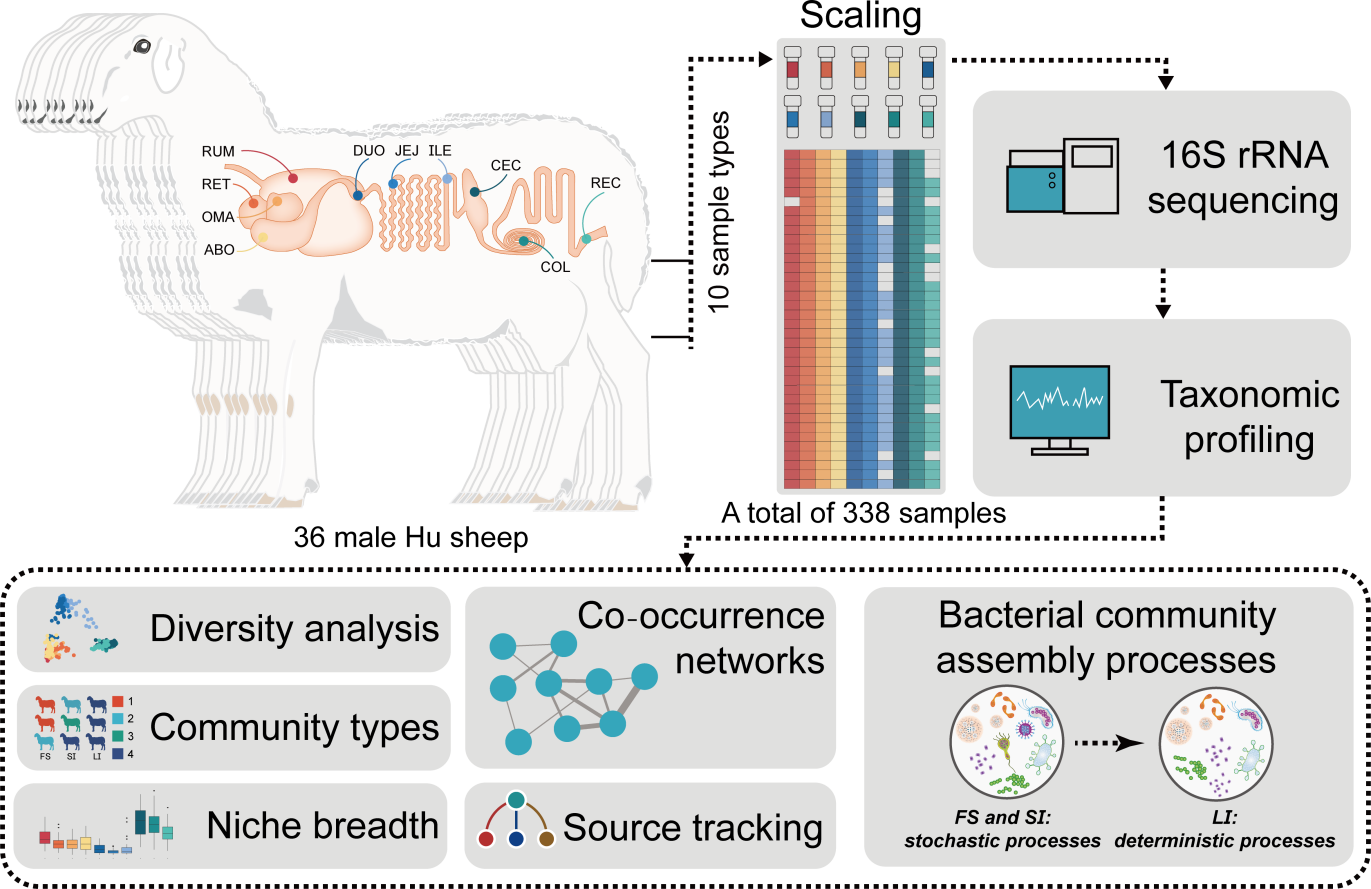
Study design and workflow.

All animal experiments and procedures were conducted under the approval and guidance of the Animal Ethics Committee of Lanzhou University (Nos.: 2020-01). A total of 36 male Hu lambs were used for this experiment (Fig. 1). All individuals were born on the same day and raised in the same sheep farm (the Minqin experimental farm of Lanzhou University, N38°43′41″, E103°013′). They were kept in individual feeding pens until the commercial slaughter age of 6 months (180 days). During the rearing period, all sheep were managed using uniform management methods, including maintaining consistent temperature and humidity levels (with an annual average temperature of 5°C–18°C and annual precipitation of 100–130 mm), feeding them the same diet (Table S1) in accordance with the recommended feeding standards for Chinese sheep (NY/T816-2004), and providing them with *ad libitum* access to water. All sheep were euthanized and slaughtered at 180 days of age, and fresh contents (20 g) were collected from the rumen (RUM), reticulum (RET), omasum (OMA), abomasum (ABO), duodenum (DUO), jejunum (JEJ), ileum (ILE), cecum (CEC), colon (COL), and rectum (REC). Samples were immediately placed in liquid nitrogen and then transferred to a −80°C ultra-low temperature freezer for storage. The dissection and sampling procedures were performed by a professional veterinarian.

### DNA extraction, PCR amplification, and Illumina NovaSeq sequencing

The frozen rumen contents stored in cryotubes were thawed at 4°C and DNA was extracted using the EasyPure Stool Genomic DNA Kit purchased (TransGen Biotech, EE301-01, Beijing, China), following the instructions provided with the kit. DNA was then assessed using 1% agarose gel electrophoresis and NanoDrop 2000 spectrophotometry. Due to insufficient volume of contents, one rumen sample, 10 ileal samples, and 11 rectal samples were excluded from the study. A total of 338 samples from 10 GIT regions of 36 sheep were obtained (see Table S2 for details). The extracted DNA was subsequently used for PCR amplification and high-throughput sequencing.

The V3-V4 region of the bacterial 16S rRNA gene was amplified using bacterial-specific barcoded primers 341F and 806R (341F: CCTAYGGGRBGCASCAG and 806R: GGACTACNNGGGTATCTAAT). The PCR system was 20 µL, including 5× Buffer 5.0 µL, dNTPs (2.5 mmol·L^−1^) 2 µL, each primer (5 µmol·L^−1^) 0.8 µL, 10 ng template DNA, and ddH2O was added to 20 µL. The PCR conditions were as follows: 3 min at 95°C, 30 s at 95°C for 27 cycles, 30 s at 55°C, 45 s at 72°C, and 10 min at 72°C. The PCR products were identified by 2% agarose gel electrophoresis containing ethidium bromide, and purified and recovered using a PCR purification kit. The recovered PCR products were quantified and sent to Tianjin Novogene Bioinformatics Technology Co., Ltd. for library construction and NovaSeq sequencing using the Illumina NovaSeq 6000 platform.

After assigning the raw sequences to their respective samples based on unique barcodes, FLASH (17) was utilized for merging the paired-end reads, followed by quality filtering using FastQC (18) using default run parameters. The filtered sequences were then demultiplexed using QIIME2 (19) (https://qiime2.org), and the DADA2 algorithm (20) was employed for constructing the amplicon sequence variant (ASV) table. Taxonomic classification of bacterial 16S ASVs was performed using the SILVA database v138 as a reference. A total of 19,399 ASVs were obtained, which were further assigned to 777 bacterial genera after excluding archaea and unassigned taxa (assigned to 42 phyla, 93 classes, 203 orders and 351 families).

### Microbial community diversity

The *vegan* R package (21) was utilized to calculate four α-diversity indices, including Pielou’s evenness, observed richness, Shannon diversity, and chao1 index. One-way ANOVA was performed to test the significance of the effect of different GIT regions on microbial diversity indices. LSD test was used to analyze the differences in microbial α-diversity among different GIT regions. Principal coordinate analysis (PCoA) and non-metric multidimensional scaling (NMDS) were employed based on Bray-Curtis distance to evaluate the compositional differences of microbial communities among different GIT regions and assess their β-diversity. Statistical analysis of β-diversity was performed using permutational multivariate analysis of variance (PERMANOVA) and analysis of community similarities (ANOSIM). The results were visualized using the *ggplot2* R package (22). The *Upset* R package (23) was used to analyze the intersection of ASVs, bacterial genera, and KEEG Level 3 among the 10 GIT regions. The intersection of ASVs and bacterial genera among the four-chambered stomach, small intestine, and large intestine was compared using an online Venn tool (http://www.ehbio.com/test/venn).

### Enterotyping analyses

The DirichletMultinomial R package was used to perform community typing analysis of bacteria in the 10 GIT regions using a Dirichlet multinomial mixture (DMM) model based on the sequencing reads at the genus level, to determine how many different enterotypes are present in each GIT region (24, 25).

### Microbial co-occurrence network analysis

The *ppcor* R package was used to construct microbial co-occurrence networks among bacteria in different GIT regions using Spearman partial correlation, and the results were visualized using *Gephi* soft (26) version 0.9.2 to explore significant relationships among bacteria in the 10 GIT regions. All correlation relationships with FDR < 0.05 were considered significant (16). The *vegan* and *igraph* R packages (27) were used to evaluate various network topology parameters, including the number of vertices, number of edges, clustering coefficient, average betweenness, and average separation. We defined edges that only appeared in one GIT region network as specialist edges, while edges that appeared in more than one GIT region network were defined as generalist edges. Key taxa were those with high connectivity in the microbial community, which had a significant impact on the composition and structure of the microbiome. In addition, we counted the number of positive and negative correlation types in each GIT region network to assess the complexity of the microbial network.

### Microbial source tracking analysis and bacterial community assembly analysis

To estimate the contribution ratio of upper GIT region microbiota to the formation of lower GIT region bacterial communities, we employed the Fast Expectation-mAximization microbial Source Tracking (FEAST) algorithm (28). The average ecological niche width of each sample was determined using the *niche.width* function in the *spaa* R package (29). To reveal differences in microbial assembly mechanisms between different GITs, *picante* v1.8.2 (30) and *NST* v3.1.10 packages in R (31) were used to calculate parameters such as the mean nearest taxon distance (MNTD), β-mean nearest taxon distance (βMNTD), and β-nearest taxon index (βNTI) to assess the phylogeny between samples. The βMNTD is used to characterize changes in phylogeny, while the βNTI is the standard deviation calculated based on the βMNTD observations and the random values obtained from the null model. A βNTI value greater than 2 or less than −2 indicates that the community assembly process is deterministic (variable selection and homogeneous selection). By contrast, when the |βNTI| value is less than 2, community assembly is dominated by stochastic processes. To further determine the classification of stochastic processes, Bray-Curtis-based Raup-Crick (RCbray) was calculated using the *vegan* v2.6.4 package. RCbray values greater than 0.95, |RCbray| values less than 0.95, and RCbray values less than −0.95 represent a dispersal limitation acting with drift, undominated processes, and homogenizing dispersal, respectively.

## RESULTS

### Bacterial diversity and composition among 10 sheep GIT regions

**Fig 2:**
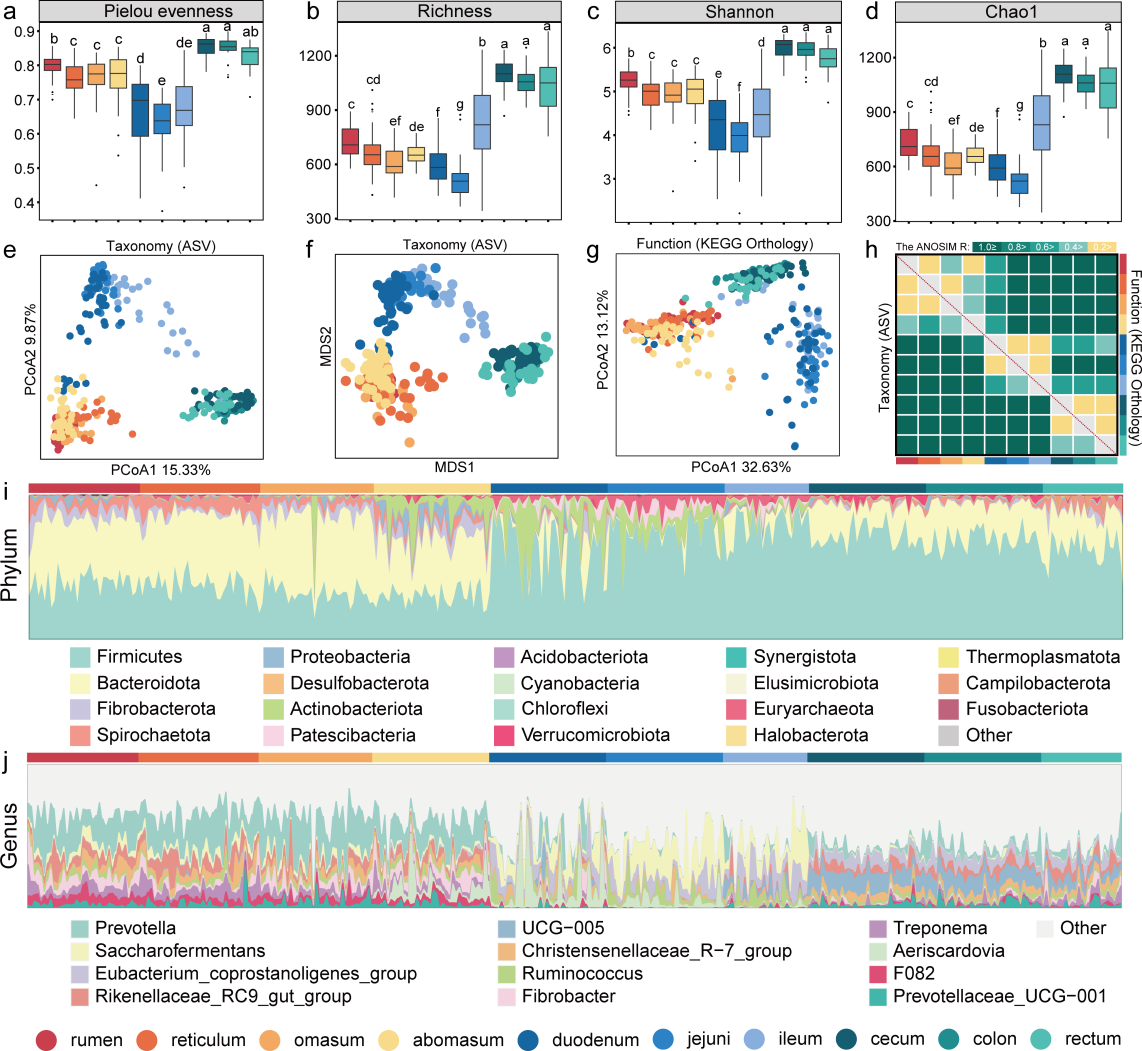
Bacterial diversity and composition among 10 sheep gastrointestinal regions. (**a–d**): Diversity of bacteria in the 10 gastrointestinal tract regions of Sheep: Calculation of Pielou, Richness, Shannon, and Chao1 Indices Based on ASV Data. (**e–g**) PCoA plot of Bray-Curtis dissimilarities based on ASVs and KEGG Ortholog (KO). Different colors represent different sheep GIT regions. (**h**): Bray-Curtis dissimilarities were assessed by analysis of similarity (ANOSIM). (**i and j**): Relative abundance of microbiota in the 10 GIT sites at the phylum and genus levels.

To illustrate the spatial heterogeneity of the bacterial community in sheep GITs, we first evaluated bacterial diversity. Overall, bacterial alpha diversity of the sheep GIT decreased gradually after the rumen, reached its lowest point in the jejunum and then began to increase, reaching stable and highest levels in the large intestine (LI), particularly in the cecum (Fig. 2a through d; Table S3). In the four-chambered stomach (FS), the rumen exhibited the highest species evenness and richness, followed by the reticulum (*P* < 0.05). In the small intestine (SI), the ileum had the highest diversity, followed by the duodenum (*P* < 0.05). We employed PCoA and NMDS to assess beta diversity. It is apparent that the bacterial flora can be primarily divided into three distinct GIT compartment groups (FS, SI, and LI) based on both taxonomic (ASV) and functional levels (KEGG Orthology) while showing a transition at the boundaries between FS to SI and SI to LI (Fig. 2e through g). The spatial heterogeneity of the sheep GIT bacterial community was confirmed by PERMANOVA and ANOSIM with a pseudo-F value of 21.08 (*P*  =  0.001) and an R-value of 0.77 (*P*  = 1e−04), respectively (Fig. 2h; Tables S4 to S6). For example, *Proteobacteria* (mean abundance = 1.20%) was the dominant bacterial phylum in the FS region; *Actinobacteriota* (11.48%), *Euryarchaeota* (5.02%), and *Patescibacteria* (3.46%) were prevalent in the SI; and *Verrucomicrobiota* (1.75%) had high relative abundance in the LI (Fig. 2i; Table S7). At the genus level (Fig. 2j; Table S8), *Prevotella* was much more abundant in the FS (22.79%) than in the SI (2.91%) and LI (5.07%), while *Saccharofermentans* was the dominant genus in the SI (16.19%), at markedly higher abundance than in the FS (3.74%) and LI (0.78%); *Oscillospiraceae UCG-005* was also considerably more abundant in the LI (10.72%) than in the FS (0.18%) and SI (1.90%).

### Bacterial enterotypes in 10 sheep GIT regions

**Fig 3:**
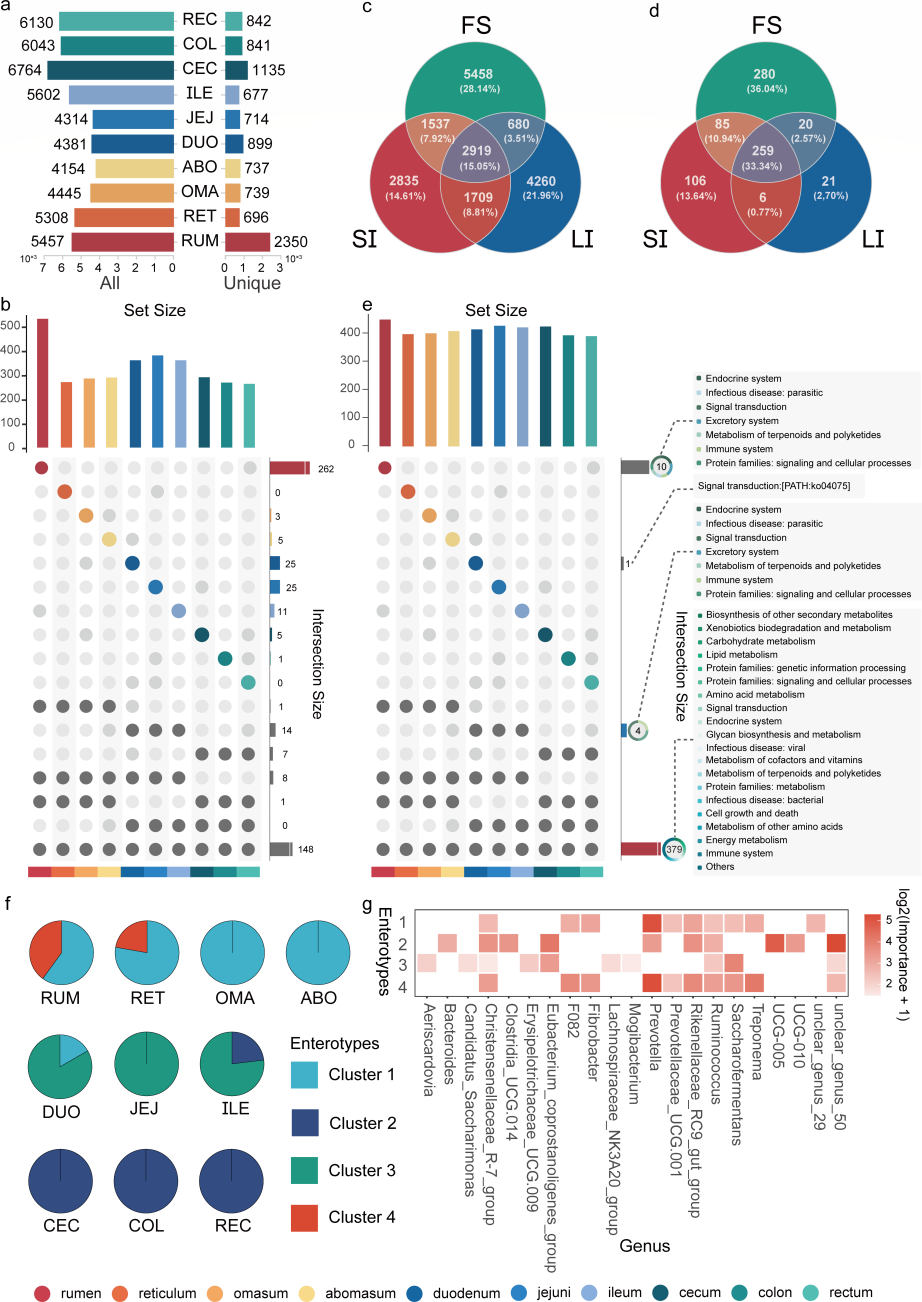
(a) Total and unique ASV number of bacteria in the ten gastrointestinal tract regions of sheep. (b) Total and unique genera number of bacteria in the ten gastrointestinal tract regions of sheep. (c) Total and unique ASV number of bacteria in the four-chambered stomach, small intestine, and large intestine of sheep. (d) Total and unique genera number of bacteria in the four-chambered stomach, small intestine, and large intestine of sheep. (e) Comparison of the levels of functional pathways (KEGG Level3) of the microbiome across regions of ten sheep GIT. Light gray boxes indicate the KEGG Level2 pathways to which these pathways belong. (f) Dirichlet multinomial mixture (DMM) model to classify the enterotypes of different GIT regions. (g) Importance of Bacterial Genera for Each enterotype (Top 5).

To investigate the commonalities and specificities of bacterial communities in the sheep GIT, we first compared the shared and unique microbial taxa (ASVs and Genera) and functional modules (KEGG Level 3) in different GIT regions. Overall, the number of total ASVs and unique ASVs followed a biogeographical pattern of variation similar to alpha diversity, with the highest number of ASVs found in the cecum (Fig. 3a). At the genus level, the rumen had the highest number of total bacterial genera and unique bacterial genera (524 and 262, respectively), with the number of unique bacterial genera far exceeding other GIT regions (Fig. 3b; Table S9). Comparing the four-chambered stomach, small intestine, and large intestine, we found that the three regions shared 2919 ASVs (Fig. 3c) and 259 bacterial genera (Fig. 3d; Table S10). The four-chambered stomach had the highest number of unique ASVs and unique bacterial genera. The large intestine had a higher number of unique ASVs than the small intestine, while the small intestine had a higher number of unique bacterial genera than the large intestine. Regional enrichment for KEGG L3 functions followed a similar pattern above, with 436 total KEGG Level-3 pathways identified in the rumen, 414 in the jejunum, and 378 in the rectum (Fig. 3e). In total, 379 pathways, predominantly related to metabolism, were shared among the ten GIT regions. It warrants mention that 10 KEGG Level-3 functions were exclusively enriched in sheep rumen, including pathways related to the endocrine system, excretory system, immune system, parasitic infectious disease, metabolism of terpenoids and polyketides, signaling and cellular process-related protein families, and signal transduction (Table S11). These results collectively illustrated the spatial heterogeneity among different GIT regions, with the greatest taxonomic and functional enrichment associated with the rumen.

Furthermore, we used a Dirichlet multinomial mixture (DMM) model to classify the enterotypes of different GIT regions. We divided the bacterial communities into four different community types (Fig. 3f). *Ruminococcus*, *Rikenellaceae RC9 gut group*, *Prevotella*, and unclear_genus_50 (members of *Lachnospiraceae*) dominated the enterotypes (Fig. 3g; Table S12). Enterotypes 1 and 4 were present in the rumen and reticulum, while only type 1 was present in the omasum and abomasum. Enterotype 3 was dominated in the three regions of the small intestine, with enterotypes 1 and 2 mixed in the duodenum and jejunum. Enterotype 2 was present in all three regions of the large intestine.

### The bacterial co-occurrence networks in 10 sheep GIT regions

**Fig 4:**
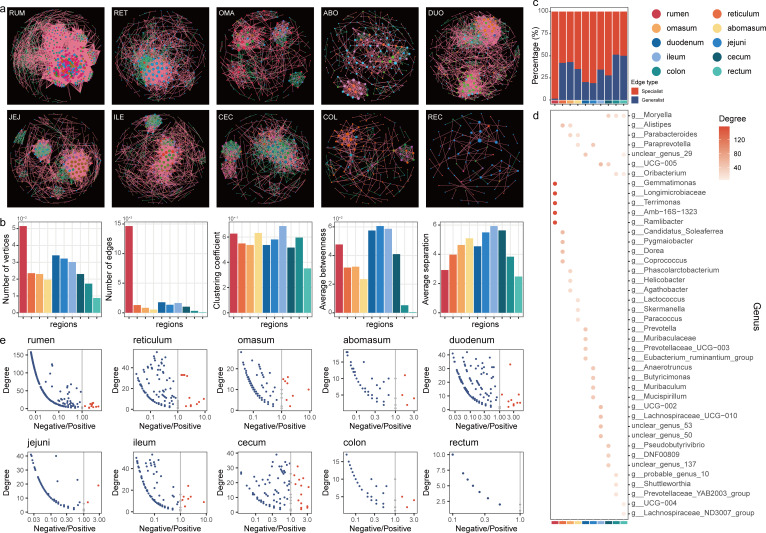
(a) Co-occurrence interaction network of the ten sheep GIT regions based on Spearman correlation indices calculated from the abundances of genera in each sample. Node colors represent different genera, while edges are colored based on positive (pink) or negative (green) correlations. (b) The topological properties of the cooccurrence networks. (c) Proportions of generalist edges and specialist edges in the 10 GIT microbial networks. (d) The top 10 key taxa in each of the 10 GIT regions are represented by colored points, with the color indicating the mean degree of each taxon. Blank positions indicate that the taxon is not considered a key taxon based on its degree in this network. (e) A scatter plot is presented for each taxon in different gut segments (duodenum, jejunum, ileum, and cecum), showing the log-transformed (log10) ratio of negative to positive interactions against degree. Red nodes indicate that the taxon has more negative interactions than positive interactions, while blue nodes indicate that the taxon has more positive interactions than negative interactions.

We calculated the correlation between bacterial communities in each GIT region at the genus level and then constructed microbial co-occurrence networks for 10 regions, analyzing the topological properties of each network (Fig. 4a). The topological characteristics of the 10 networks were different (Fig. 4b; Table S13). The rumen bacterial co-occurrence network had more vertices (*n* = 515) and edges (*n* = 14,648) than the other nine GIT regions, with the duodenum coming in second. The clustering coefficient of the ileum microbial co-occurrence network (0.69) was higher than that of the other nine GIT regions, and it is noteworthy that the clustering coefficients of the rumen (0.63) and abomasum (0.63) were also greater than 0.6. The co-occurrence network of the small intestine microbial community showed a high average betweenness centrality (589.14) and average separation (5.32). These results indicate that the rumen bacterial community exhibits a more complex microbial network structure, with more vertices, edges, clustering coefficients, average betweenness centrality, and lower average path length values. The colon and rectum had a relatively high proportion (51% and 50%, respectively) of generalist edges (present in at least 2 GIT regions), while the rumen contained the greatest number of specialist edges (present in only one GIT region), indicating that as the number of unique microbes increases, many new microbial relationships are established in the rumen (Fig. 4c).

Next, we investigated the key taxa (highly connected taxa) in the 10 co-occurrence networks. Figure 4d shows the top five vertices with the highest degree in each network (a total of 42 genera; Table S14). *Longimicrobiaceae*, *Candidatus Soleaferrea*, *Parabacteroides*, *Lactococcus*, *Prevotella*, *Anaerotruncus*, *UCG-002*, *Pseudobutyrivibrio*, *Oribacterium*, and *Moryella* were the key taxa in the rumen, reticulum, omasum, abomasum, duodenum, jejunum, ileum, cecum, colon, and rectum, respectively. *Moryella*, *Alistipes*, *Oribacterium*, *Parabacteroides*, *Paraprevotella*, *UCG-005*, and unclear_genus_29 were the key taxa in more than one co-occurrence network. It is noteworthy that 12 genera among these key microbes belong to the *Lachnospiraceae* family, highlighting their important role in the sheep GIT. We further analyzed the positive and negative correlations between genera in each network. Interestingly, the vast majority of genera, including all key taxa, had more positive correlations (1,867) with other genera than negative correlations (199; Fig. 4e; Table S15). Only a few genera had more negative correlations than positive correlations with other microbes, such as *Prevotellaceae UCG-003*, *Rikenellaceae RC9 gut group*, *Prevotellaceae NK3B31 group*, *Butyrivibrio*, and *Lachnospiraceae ND3007 group* in the rumen.

### The bacterial community assembly among 10 sheep GIT regions

**Fig 5:**
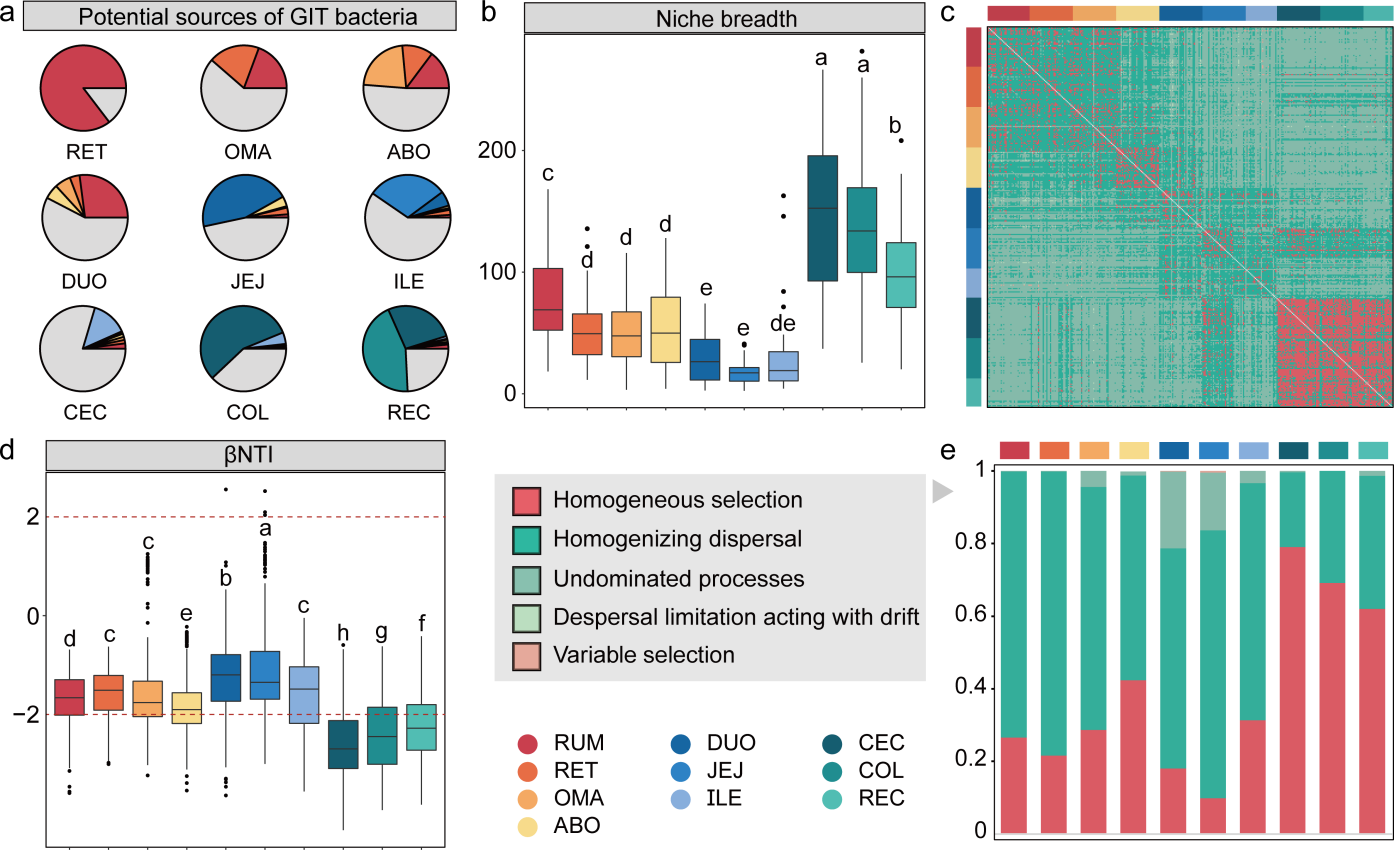
(a) Potential sources of each bacteria. The pie chart illustrates the contribution of the upstream GIT region to the source of bacteria in the region. (b) 10 sheep GIT region niche breadth of the bacterial communities. (c) Ecological processes between paired samples. (c) The values of the weighted beta nearest taxon index (βNTI) for 10 sheep GIT bacterial communities. The βNTI distributions differed significantly across the sheep GIT [F_Welch_(9,2071.00) = 273.39, *P* < 0.0001], from stochastic assembly (|βNTI|  <  2) to deterministic community assembly (|βNTI|  >  2). (d) The percentage of the turnover in 10 GIT bacterial community assemblies.

The spatial heterogeneity of the sheep GIT bacterial community has prompted us to further investigate the influence of microbiota in the upstream GIT regions on those in the downstream GIT regions. To this end, we employed FEAST to quantify the microbial sourcing patterns of the nine GIT regions following the rumen. Our results showed that the bacterial community in the rumen had a sustained impact on the bacterial communities in all nine downstream GIT regions, with an average contribution of 17% (Fig. 5a; Table S16). The rumen microbial community had the highest contribution to the reticulum microbial community at 85.36%, while the colon microbial community had the lowest contribution at 0.57%. In addition, we found that the bacterial communities in the colon and rectum were strongly influenced by those in the cecum, although the sourcing patterns of 80% of the bacterial community in the cecum were unclear. Further comparison of the ecological niche breadth revealed that the bacterial community in the cecum had the highest ecological niche breadth (Fig. 5b; Table S17), indicating a lower degree of specialization and a higher adaptability and flexibility to utilize various types of resources.

Furthermore, we utilized ecological models to investigate the internal drivers of the formation of bacterial microbial communities in each GIT region. We first determined the distribution of the weighted bacterial community assembly (βNTI) values for all sample sites (Fig. 5c), which revealed significant differences among regions across the whole GIT [F_Welch_ (9,2071.00) = 273.39, *P* = 0e + 00; Fig. 5d], from stochastic assembly (|βNTI|  <  2) to deterministic community assembly (|βNTI|  >  2). We next used an identity-based null model to further differentiate the processes driving community structure for different GIT regions (Fig. 5e; Table S18). The stochastic process of homogenizing dispersal (HD) contributed 56.24% to GIT bacterial community assembly, followed by the deterministic process of homogeneous selection (HS, 38.88%). There are significant differences in the assembly processes of bacterial communities in different GIT regions (*P* < 0.05). The stochastic process of HD was mostly responsible for the assembly and turnover of the bacterial communities of the four-chambered stomach and small intestine (relative contribution, 67.77%). Here, undominated processes (UP) also exert a certain influence on the assembly of bacterial communities in the small intestine. In the large intestine regions, the deterministic processes of HS contributed up to 70.08% to the bacterial community assembly of the large intestine, followed by HD (29.37%).

## DISCUSSION

The rumen microbiota has a close relationship with the nutrition of ruminants, and the species, activity, and abundance of microbiota affect the digestive and metabolic functions of the rumen, especially since rumen microbiota fermentation provides over 70% of the host’s energy (9). Therefore, humans have long focused on rumen microbiota and generally believed that rumen microbiota diversity is the richest in the entire digestive system of ruminants. However, we found that the bacterial diversity and evenness in the rumen of sheep are lower than those in the three regions of the large intestine. Based on the niche breadth, this may be because bacterial taxa in the three regions of the large intestine can utilize more diverse resources, resulting in lower species specialization and more generalization (32). On the other hand, the U-shaped distribution of diversity may correlate with the physiological functions of different GIT segments, such as the rate of food passage, environmental pressures, and microbial metabolism. Abundant substrates and both vertical and horizontal transfers of microbes enhance the diversity of the ruminant’s stomach chamber microbial communities. The small intestine, primarily designed for nutrient absorption with villi and microvilli structures optimized for efficient nutrient uptake, may not be conducive to the proliferation of diverse microbial communities. By contrast, the slower movement of chyme in the large intestine, particularly in the cecum, provides microbes with a longer residence time, favoring the stability and development of microbial communities. The number of unique bacterial species in the rumen is much higher than that in other GIT regions, both at the ASV and genus levels. These taxa provide the rumen with more unique functions in macro and micronutrient digestion and absorption. Consistent with the results of other sheep breeds such as Ujumqin sheep (33) and Aohan fine-wool Sheep (34), we also found that the most significant spatial heterogeneity of GIT regions comes from the four-chambered stomach, small intestine, and large intestine, which can be divided into these three parts based on both abundance composition and community structure. As the relatively upstream region of the entire digestive tract, the four-chambered stomach continuously ingests external microorganisms from the diet and environment, and compared with the small and large intestines, the four-chambered stomach has more bacterial groups and unique groups.

“Enterotypes” are indicators used to distinguish the composition of the microbiota in the gut, similar to blood types (24). They are the strongest distinguishing factor for gut microbiota community structure among individuals, providing an attractive framework for understanding the impact of ecological constraints and community characteristics on the community structure of various GIT regions (35). We found that the GIT microbiota enterotypes of sheep exhibit obvious regional specificity, and the changes in enterotypes in cross-sectional samples can be used to identify changes in the sheep microbiota community. Previous studies (36) have shown that the rumen microbiota composition of goats can be divided into two enterotypes dominated by *Prevotella* and *Ruminococcus*, respectively. Similarly, this study found that two enterotypes dominated by *Prevotella* also dominate the microbial community in the four four-chambered stomachs of sheep, emphasizing the ability of sheep four-chambered stomach microbiota to degrade plant fiber and produce propionic acid (5). Although we did not find that *Ruminococcus* dominates any enterotype, it has a high contribution to all four enterotypes. The enterotype dominated by *Saccharofermentans* dominates the microbial community composition in the small intestine of sheep. *Saccharofermentans* contains multiple carbohydrate-activating enzymes, which enable it to ferment various substrates and produce acetic and propionic acids (9, 37). This means an enhancement in the digestive capacity of sheep small intestine enzymes. The enterotypes in the large intestine of sheep are relatively stable, and all individuals have enterotype 2 dominated by unclear_genus_50 (members of *Lachnospiraceae*) and UCG-005 (members of *Oscillospiraceae*). These community combinations may help further ferment various nutrients in food residues in the large intestine, especially crude fiber. The classification method based on enterotypes will have an impact on personalized feeding in terms of nutrition and microbiota intervention.

Consistent with other ruminants, our study shows that the bacterial co-occurrence network in the sheep rumen is the most complex, and nearly 98.5% of the interaction between rumen bacteria exists only in the rumen. Bacterial interactions in the duodenum are more complex than those in the jejunum and ileum, and similar phenomena have been found in the gut microbiota of pigs (38) and chickens (16). The differentiation of bacterial niche breadth in the large intestine may reduce direct competition between different types of bacteria, thereby reducing their interactions. Compared with bacteria in the four-chambered stomach and small intestine, which are exposed to more digestive fluids and mechanical movements, the relatively stable environment of the large intestine (slow flow rate, neutral to slightly acidic pH) may lead to a relatively stable bacterial community structure, resulting in fewer microbial interactions (33, 34). Microbes in the GIT usually form symbiotic relationships, promoting each other’s growth and metabolism, thereby maintaining the stability and balance of the entire microbial community (39). This may explain why there are fewer negative correlations.

The differences in the assembly processes of microbes caused by different GIT regions lead to differences in the microbial community composition among sheep GIT microbes (33). Stochastic processes dominate the formation of most bacterial communities in the four-chambered stomach and small intestine, while deterministic processes drive the formation of bacterial communities in the large intestine. Under the same feeding and management conditions, the turnover of bacterial communities in the large intestine is mainly caused by homogenous environmental selection pressure. In the large intestine, food residues stay longer, and microbes have more time and opportunities to grow and reproduce in this relatively stable environment (40). Therefore, the assembly of microbial communities may be more influenced by local environmental factors and tend to be deterministic. By contrast, food bolus has a lower average residence time in the four-chambered stomach and small intestine, and bacteria are less affected by environmental selection. Therefore, the directed flow of food bolus between the four-chambered stomach and small intestine has a higher spreadability for bacterial communities in the four-chambered stomach and small intestine, resulting in a lower turnover rate of communities (33, 41). The results of source tracking analysis support this result, and the microbial composition of food bolus changes significantly after entering the cecum. The average residence time of food bolus in the sheep’s small intestine is only 1.5–4.5 hours, and microbes that cannot form close contacts and settle in a short time will enter the large intestine for further reproduction (33). The higher ecological niche diversity and mild habitat of the large intestine provide abundant nutrition and living space for bacteria, and the interactions between microbes weaken, attracting a large number of microbes that cannot settle in the four-chambered stomach and small intestine to gather and live. In the future, more research focusing on *in vitro* simulation analysis is needed to determine the environmental factors that may affect the deterministic processes of the large intestine bacterial community. Source tracking analysis emphasizes the important role that cecal microbiota may play in the health and productivity of sheep, but relevant research is still lacking and deserves more attention in the future.

### Conclusion

We conducted an assessment of the bacterial community composition and assembly mechanisms in 10 GIT regions of 36 Hu sheep under identical feeding conditions. Microbial diversity, species composition, function, gut type, and microbial interactions analysis demonstrated the spatial heterogeneity of bacterial colonization along the gastrointestinal tract. Bacterial diversity showed a “U”-shaped distribution from the rumen to the rectum, with the highest bacterial diversity in the cecum. The rumen had the highest number of bacterial genera and unique bacterial genera, while the rectum had the least. The complexity of bacterial community networks and interactions between taxa decreased from the four-chambered stomach to the large intestine, with more positive correlations between microbes. The three parts of the large intestine had higher ecological niches, and deterministic processes drove the formation of bacterial communities in the large intestine, while the formation process of bacterial communities in the four-chambered stomach and small intestine was more stochastic.

## Data Availability

Raw data obtained by 16S rRNA sequencing were uploaded to the NCBI sequence read archive under BioProject ID PRJNA785332.

## SUPPLEMENTAL MATERIAL

Supplemental tables spectrum.01110-24-s0001.xlsx Tables S1 to S18.

ASM does not own the copyrights to Supplemental Material that may be linked to, or accessed through, an article. The authors have granted ASM a non-exclusive, world-wide license to publish the Supplemental Material files. Please contact the corresponding author directly for reuse.
